# Composition Heterogeneity and Low-Molecular-Weight Allergen Content of *Dermatophagoides farinae* House Dust Mite Allergen Extracts Used in Veterinary Medicine

**DOI:** 10.3390/vetsci12090824

**Published:** 2025-08-27

**Authors:** Marie Welters, Ana Mas-Fontao, Silvia T. Auxilia, Thierry Olivry

**Affiliations:** 1 AniCura Haaglanden Referral Hospital, Frijdastraat 20a, 2288 EZ Rijswijk, The Netherlands; marie.welters@anicura.nl (M.W.); silvia.auxilia@anicura.nl (S.T.A.); 2 Nextmune Spain, Valentin Beato 24, 28037 Madrid, Spain; ana.mas@nextmune.com; 3 Nextmune AB, Biblioteksgatan 8, 111 46 Stockholm, Sweden

**Keywords:** allergen, allergy, *Dermatophagoides*, dog, extract, IgE, serological testing

## Abstract

Extracts used for allergen sensitization testing in humans are heterogeneous in composition and may contain low levels of important allergens. In contrast, there is scant information on the variability of veterinary allergen extracts. We selected two batches of extracts of *Dermatophagoides farinae* house dust mites from each of three different manufacturers. We observed a 3.4-fold difference in total protein content between the two extracts with the lowest and highest amounts of allergens. After electrophoresis, the band migration patterns were found to be similar between the two batches of extracts from two manufacturers and dissimilar in the third; the migration profiles were heterogeneous between manufacturers. The six extracts did not contain the same concentrations of Der f 1 and Der f 2, with fold changes between the highest and lowest concentrations measured to be 14.3× for Der f 1 and 8.0× for Der f 2. This allergen composition heterogeneity resulted in differences in ELISA seropositivity when allergic dogs had low serum concentrations of IgE against Der f 1 and Der f 2. Our findings demonstrate that, like in human allergology, allergen extracts for veterinary use are also heterogeneous in their protein content and allergen composition. This heterogeneity can impact results when determining allergen sensitizations using crude extracts.

## Simple Summary

Allergy testing often uses extracts from allergens, such as house dust mites, to identify the substances that cause allergic reactions. It is well established that human allergy test extracts vary in their composition, and little is known about those used in animals. We studied two batches of house dust mite extracts from each of three veterinary manufacturers and found large differences in protein content and allergen levels. For example, one allergen, Der f 1, varied up to 14 times between the most and least concentrated extracts. We also observed that the composition of these extracts influenced their ability to detect allergic responses in dog blood samples. These findings highlight that veterinary allergen extracts can vary greatly, and this variability can impact allergy test results. Just as in human medicine, improving the consistency of extracts for veterinary allergy testing is crucial for accurately identifying what sensitizes pets.

## 1. Introduction

For decades, allergen immunotherapy (AIT) has been used as a disease-modifying intervention in allergic human and animal patients to prevent the recurrence of clinical signs upon further allergen contact [1]. To select allergens for AIT, physicians and veterinarians have long relied on a combination of specific IgE serology or skin testing using allergen extracts. Consequently, having consistent and homogeneous allergen extracts is crucial for accurately determining the patient’s sensitizations.

Unfortunately, allergen extracts have two major limitations. Firstly, those used to test allergic humans have long been known to be heterogeneous in their composition. This heterogeneity is not surprising, as crude extracts are made up from ground natural biological material that is difficult to standardize. In past years, surprisingly high variability in major allergen content has been reported for extracts of *Dermatophagoides* house dust mites (HDMs) [2,3,4], German cockroaches, molds [5,6,7], and pollens [2,8,9,10] used to test human patients. A similar variability in protein composition, assessed via polyacrylamide gel electrophoresis (PAGE), also appears to exist in allergen extracts for veterinary use [11], although this information is limited to a single report in which specific allergen concentrations were not quantified.

A second concern is that these heterogeneous extracts may contain very low levels of important allergens [2,4,5,6,7,8,9,10,12]. For example, an extract of the *Dermatophagoides farinae* (Der f) HDM would be expected to include several thousand proteins expressed from the nearly 11,000 protein-encoding genes predicted in its genome [13,14]. Currently, however, there are only 40 officially named allergens for Der f [15], meaning that its extract contains mostly irrelevant, non-allergenic proteins. A low concentration of clinically relevant allergens may yield false-negative results in both skin and serological tests, especially in allergic patients who have low levels of specific IgE (sIgE) against these allergens. Such variability in testing results could impact the selection of allergens for immunotherapy and, thus, potentially affect the efficacy of that intervention.

In humans and animals, HDMs from the *Dermatophagoides* genus are recognized as the most common source of allergens in patients with allergies [16,17,18]. In humans, the most important *Dermatophagoides* allergens are those from group 1 (Der f 1, Der p 1) and 2 (Der f 2, Der p 2), which are cysteine proteases and proteins from the NPC2 family, respectively [19]. While, at first, Der f 2 was thought to be a common allergen only in dogs from Japan, it has now been confirmed to sensitize most mite-allergic dogs worldwide [17,20,21]. Similarly, Der f 1 was initially considered an allergen uncommonly targeted by sIgE in atopic dogs [20,22,23,24]. Yet, a recent study has confirmed that it may also be relevant to mite-sensitized dogs [25]. Whereas the high-molecular-weight allergens Der f 15 [26], Der f 18 [27], and Zen-1 [23,28] were first believed to be the most important Der f allergens in dogs, we recently showed that their targeting by IgE mainly represents a glycan-mediated cross-reactivity with the secreted mucins of the ubiquitous nematode *Toxocara canis*; this discovery casts some doubt on the clinical relevance of sensitization to these high-molecular-weight allergens in dogs [29].

We hypothesize that commercial extracts of Der f for veterinary use, like those used in humans, are also heterogeneous in their protein composition and might contain variable and perhaps low amounts of major allergens.

In this study, we compared the protein composition and the concentration of Der f 1 and Der f 2 in several commercial extracts of Der f HDMs. Furthermore, we investigated the potential consequences of any variability in allergen content on IgE serological results obtained with these extracts.

## 2. Materials and Methods

### 2.1. Animals

We selected 21 archived sera from dogs suspected of having allergic disease(s) (any type) by their veterinarians; detailed clinical information was not available for these pets. In these dogs, allergen-specific IgE serology with the Pet Allergy Xplorer (PAX; Nextmune, Stockholm, Sweden) [30] had revealed variable levels of sIgE against Der f 1 and Der f 2. There were seven dogs with low levels of sIgE (Class 1: 28.00 to 99.99 ng/mL) against Der f 1 (Dogs 1 to 3) or Der f 2 (Dogs 10 to 13). Five others had medium levels (Class 2: 100.00 to 399.99 ng/mL) of sIgE against these allergens (three for Der f 1: Dogs 4 to 6, and two for Der f 2: Dogs 14 and 15). Six additional dogs had high levels of sIgE (Class 3 or 4: 400 ng/mL and above) against Der f 1 (Dogs 7 to 9) or Der f 2 (Dogs 16 to 18). Finally, the last three dogs had very low levels of sIgE (<28.00 ng/mL) against both Der f 1 and Der f 2 in the PAX (Dogs 19 to 21). Table S1 contains the sIgE levels against Der f and *D. pteronyssinus* (Der p) extracts and their molecular allergens obtained from PAX testing.

### 2.2. Allergen Extracts

We assembled two batches (numbered 1 and 2) of Der f extracts, each produced in different years by two distinct manufacturers of allergens for animal use (laboratories A and B). For comparison, we obtained two batches from a company producing extracts for both human and veterinary use (laboratory C). Extracts from laboratories A and B were directly obtained from the manufacturer; extracts from laboratory C were purchased commercially.

### 2.3. Protein Content Determination

The protein content of each of the six Der f extracts was determined in duplicate using the Bradford assay (Bio-Rad Laboratories, Hercules, CA, USA), according to the manufacturer’s instructions, with a bovine serum albumin curve as the standard to interpolate using linear regression (InStat, GraphPad Software, San Diego, CA, USA). The values reported represent the average of the two values.

### 2.4. Sodium Dodecyl Sulfate–Polyacrylamide Gel Electrophoresis (SDS-PAGE)

The proteins of the six Der f extracts were separated on an in-house casted 15% polyacrylamide gel containing SDS under non-reducing conditions. We loaded 6 µg of protein in each lane. In addition, 2 µg of native purified Der f 1 (InBio, Cardiff, UK) and recombinant Der f 2 (Angany Innovation, Val de Reuil, France) were run on the two rightmost lanes as allergen standards. The sizes of the protein bands were compared with the Dual Colour Precision Plus Protein Standard (BioRad Laboratories, Hercules, CA, USA). The electrophoresis was run for approximately 1 h at 120–140 V using the Mini-PROTEAN Tetra-Cell system (BioRad Laboratories, Hercules, CA, USA). After this step, the gel was flushed with distilled water, and the bands were stained with Coomassie solution overnight at 4 °C with gentle shaking. The following day, the gel was destained with a methanol–acetic acid solution at room temperature, with shaking, and the solution was changed every 15 min until a clear background with distinct bands was observed.

### 2.5. Der f 1 and Der f 2 Quantification

The concentrations of Der f 1 and Der f 2 were determined in single replicates in all Der f extracts using the kits EPC-DF1-1 and EPC-DF2-1 (InBio, Cardiff, UK), respectively, after normalizing all extracts to their total protein content. Final concentrations were calculated after interpolating results in the obtained standard curves using a four-parameter logistic curve fit (x-axis plotted on a logarithmic scale), as suggested by the manufacturer. Concentrations of the high-molecular-weight allergens Der f 15, Der f 18, and Zen-1 were not determined due to the lack of commercially available antibodies and immunoassays.

### 2.6. ELISA

Each one of the six Der f extracts was coated overnight at 4 °C at a concentration of 0.25 µg of protein per well in carbonate buffer (pH 9.6). The following day, the wells were washed three times with 250 µL of washing buffer per well (1× Tris-buffered saline [TBS]/0.05% Tween 20). Subsequently, the wells were blocked with the blocking buffer (1× TBS/0.5% polyvinyl pyrrolidone [PVP]-10) for 2 h at room temperature. After blocking, the wells were washed once with the buffer before adding, in duplicates, 50 µL of canine sera diluted 1:10 or the diluent buffer alone (1× TBS/0.05% Tween 20) as a negative control. The sera were incubated overnight at 4 °C. The next day, the wells were washed four times with the washing buffer, and 50 µL of mouse anti-dog IgE monoclonal antibody 5.91 labeled with alkaline phosphatase (1 µg/mL) was added and incubated for 2 h at room temperature. Finally, the wells were washed six times with the washing buffer before adding 50 µL of para-nitrophenyl phosphate (pNPP) substrate (Moss, Pasadena, MD, USA). The pNPP was incubated for 30 min in the dark at room temperature, and the reaction was stopped with 50 µL of 1 N sodium hydroxide; the absorbance was read at 405 nm. The positivity threshold of 0.2 was selected as the rounded, highest value of either one of the following two calculations: the mean plus three standard deviations of the no-reagent wells (which corresponds to the limit of detection), or the mean plus three standard deviations of the duplicates of the sera from the three dogs (Dogs 19–21) that were IgE seronegative for the Der f extract in the PAX (i.e., non-sensitized dogs).

## 3. Results

### 3.1. Protein Content

**Figure 1:**
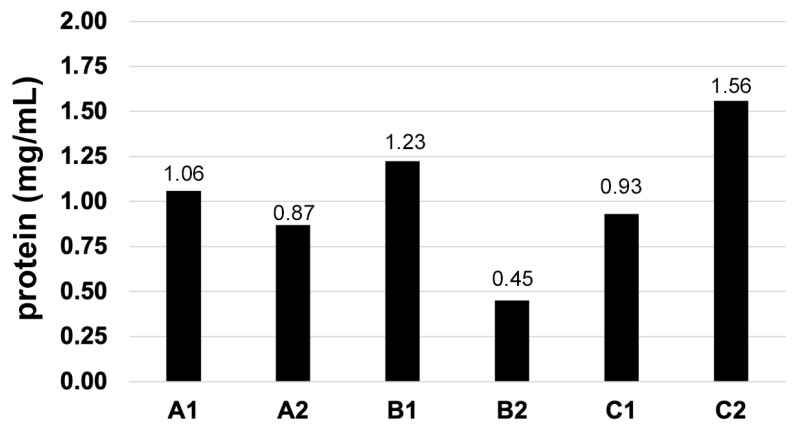
Total protein concentrations: There is heterogeneity in protein concentrations between extracts, both within and between laboratories.

First, we determined the total protein content in each of the six extracts. The protein concentrations varied between 0.45 mg/mL (extract B2) and 1.56 mg/mL (extract C2); the fold change between the lowest and highest protein contents was 3.4× (Figure 1). While the two extracts from Manufacturer A had somewhat similar protein concentrations (a difference of 1.2×), the difference was higher between the extracts of Manufacturer B (2.7×) and those of Manufacturer C (1.7×). These differences in total protein concentrations were the first indication of heterogeneity between the tested Der f extracts. All extracts were normalized based on these protein concentrations for all subsequent tests.

### 3.2. SDS-PAGE and Mass Spectrometry

**Figure 2:**
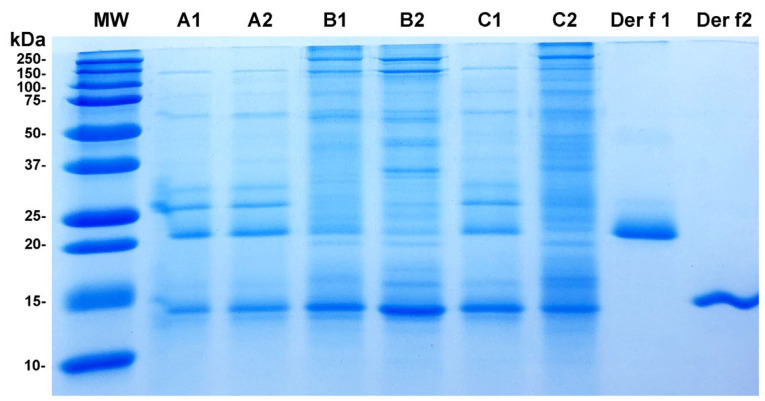
SDS-PAGE: The electrophoretic migration patterns of the two batches of Der f extract from manufacturer A (A1 and A2) were homogeneous, as were those from manufacturer B (B1 and B2). The two batches from manufacturer C (C1 and C2) were the most dissimilar. Overall, there was some heterogeneity between the extracts produced by the three manufacturers. MW: molecular weight marker lane; kDa: kilodaltons.

The visualization of the protein electrophoretic migration patterns and band thickness revealed that the extracts from manufacturers A, B, and C were quite different (Figure 2). The extracts A1 and A2 had similar patterns, with only minor visible differences in band thickness. A similar resemblance existed between extracts B1 and B2. However, the band profiles differed greatly between the two extracts from manufacturer C.

It is important to note that the mass spectrometry sequencing of the bands co-migrating with Der f 1 and Der f 2 in each extract confirmed that sometimes, these bands contained other allergens besides Der f 1 and Der f 2 (Figure S1). Therefore, comparing the thickness of these two bands between extracts alone is not an accurate way to determine the sole content of each of these two antigens.

Overall, these results confirmed the variability among extracts produced by different manufacturers. While two of the three manufacturers exhibited relatively consistent band migration patterns across batches produced years apart, this was not the case for the third manufacturer.

### 3.3. Der f 1 and Der f 2 Quantification

**Figure 3:**
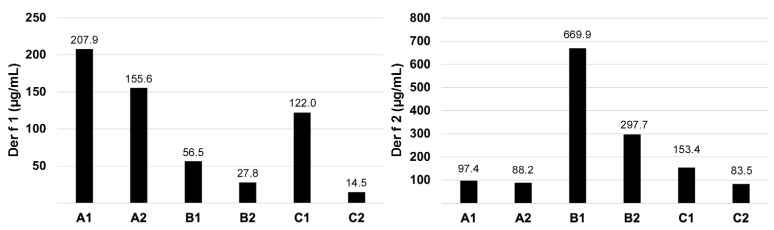
Der f 1 and Der f 2 allergen content: There was heterogeneity, both within and between manufacturers, in the concentrations of Der f 1 (**left panel**) and Der f 2 (**right panel**) in each of the six tested extracts.

The concentrations of Der f 1 (left) and Der f 2 (right) in the six extracts are shown in Figure 3. As could have been predicted from their electrophoretic migration patterns, the two extracts from manufacturer A were not too dissimilar in the content of these two allergens; they were richer in Der f 1 than Der f 2. The reverse was true for the B extracts that had more Der f 2 than Der f 1. These two B extracts differed in their content of the two allergens, with B1 containing about twice as much Der f 1 and Der f 2 as B2. The extract C1 contained eight times more Der f 1 and about twice as much Der f 2 as C2; the latter contained the least amount of these two allergens compared to the other five extracts.

Altogether, the fold changes between the highest and lowest concentrations measured were 14.3× for Der f 1 and 8.0× for Der f 2.

When adding the concentrations of both allergens, the following rankings were obtained: B1: 726.4 µg/mL, B2: 325.5 µg/mL, A1: 305.3 µg/mL, C1: 275.4 µg/mL, A2: 243.8 µg/mL, and C2: 98.0 µg/mL. Consequently, there was a greater than 7-fold variation in the combined concentrations of Der f 1 and Der f 2 between B1 and C2.

### 3.4. ELISA

The variability in allergen content between Der f extracts, exemplified by the heterogeneity in their concentrations in Der f 1 and Der f 2, had some impact on whether some sera with variable levels of IgE were positive or negative when tested by ELISA, the most used technique for routine allergen-specific IgE serology.

**Figure 4:**
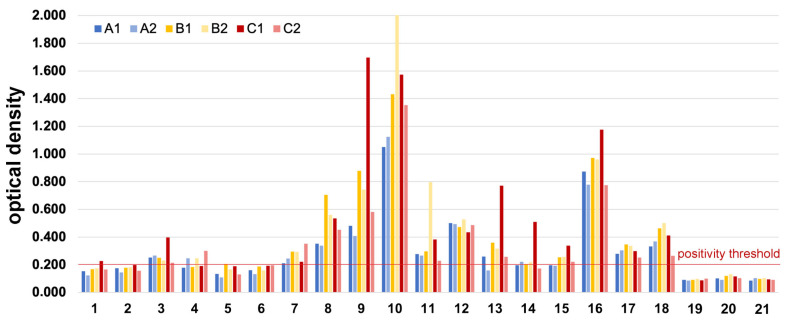
ELISA for the detection of Der f-specific IgE: The sera with no detectable Der f-specific IgE on the PAX (Dogs 19–21) were also negative (OD < 0.200) by ELISA with each of the six extracts. If dogs had low or medium levels of Der f 1-specific IgE on the PAX (Dogs 1–6), they were more likely to have a negative detection of Der f-specific IgE by ELISA. The serum level of Der f 2-specific IgE had a relatively lower impact on the probability of positivity in the Der f by ELISA.

As expected, the dogs with very low levels of sIgE against Der f 1 and Der f 2 (Dogs 19 to 21) had no detectable Der f-specific IgE when tested against the six extracts by ELISA (Figure 4).

Although the dogs tested therein were likely sensitized to additional Der f allergens and not included on the PAX, the Der f 1 content of the extract appeared to impact most of the detection of Der f-specific IgE (Figure 4). Indeed, only one of the three dogs (Dog 3) with a low level of detectable IgE against Der f 1 was positive (i.e., with an OD ≥ 0.200) with each of the six Der f extracts, while the two other dogs showed negative ELISA results with five of the six extracts. A similar pattern was seen with the dogs with medium levels of sIgE against Der f 1 (Dogs 4 to 6): only Dog 4 was found to be consistently sensitized to Der f by ELISA using half of the extracts. In contrast, when dogs had high levels of IgE against Der f 1 (Dogs 7 to 9), they were positive by ELISA using the six Der f extracts.

The dogs’ levels of sIgE against Der f 2 in the PAX also had an impact, albeit smaller, on their seropositivity to the six Der f extracts (Figure 4). The lowest reactivities, as determined by ELISA, were observed for extracts A1 and A2 in Dogs 13 to 15. Surprisingly high optical densities were observed with each extract using the serum from Dog 10, which exhibited low levels of Der f 2-specific IgE (28.28 ng/mL; see Table S1). Since this dog did not show any detectable IgE against the other Der f and Der p allergens on the PAX, we speculate that the dog’s serum contained IgE against one or more Der f allergens not included in the PAX macroarray.

Altogether, these results confirm that the allergen profile and content of an extract directly influence whether atopic dogs’ sera with varying levels of IgE to the major allergens Der f 1 and Der f 2 will be detected as having Der f-sIgE when tested by ELISA.

## 4. Discussion

This study confirmed that crude allergen extracts used in veterinary medicine vary in their protein content and composition. These inconsistencies, like those observed in human medicine, raise concerns about the reliability of serological and intracutaneous sensitization detection tests that rely on crude extracts. Consequently, the effectiveness of AIT, which is based on the results of sensitization tests, could be compromised if the formulation does not include relevant allergens that are absent or present in low concentrations in the used extracts.

We first analyzed the total protein content as an initial quality control measure to investigate the degree of homogeneity between the various allergen extracts, even though this method does not differentiate between specific allergens or evaluate their immunogenic properties [12]. Our results revealed up to a 3.4-fold difference in total protein content across extracts. Despite this variation, the overall heterogeneity we observed was lower than that reported in similar studies comparing crude allergen extracts for human use. For instance, one study noted a 10-fold difference in total protein levels between the highest and lowest concentrations in nine extracts of Der p and a 50-fold difference in the range of proteins across seven extracts of *Blomia tropicalis* [4]. In another report, the maximal difference in protein concentration between eight extracts was approximately 4-fold for Der p and 17-fold for Der f [3]. Such high variability in total protein concentration is not limited to HDM extracts, as it has also been observed with those of grass pollen (an 8-fold difference) [9] and German cockroach (a 6-fold difference) [31].

As previously shown for Der f and Der p extracts in humans [3,4], the SDS-PAGE analysis of our crude Der f extracts revealed distinct patterns of protein bands, confirming the variability in their allergen composition. The protein migration profiles of the two extracts from veterinary manufacturers A and B were relatively similar to each other but differed between manufacturers. An important limitation of gel electrophoresis is that one cannot assume that each band represents the migration of a single protein. In fact, as shown in Figure S1, the bands co-migrating with Der f 1 not only corresponded to this allergen but also variably contained Der f 36. Similarly, those co-migrating with Der f 2 also contained Der f 34. Consequently, it would be inappropriate to compare the thickness of these two bands as solely indicative of their content in Der f 1 or Der f 2.

Another substantiation of the heterogeneity of Der f extracts was evident in the varying levels of two important allergens. We observed up to a 14-fold variation in the levels of Der f 1 and an 8-fold difference in the levels of Der f 2 between the tested extracts. This variability in allergen levels was not surprising, given that previous studies had reported between 4- and 35-fold variations in the concentrations of group 1 mite allergens in various HDM extracts [2,3,4]. Similarly, 3- to 41-fold differences were noted for levels of group 2 mite allergens [2,3,4].

This substantial variation in major allergens is not limited to crude extracts of HDMs, as it has also been documented in those of other organisms. For instance, an approximate 13-fold variation in Bet v 1 levels was observed among six birch pollen extracts in one study [2] and a 10-fold variation in levels of the same allergen in extracts from five different manufacturers in another [10]. Similarly, eight Timothy grass extracts showed variations of approximately 12-, 6-, and 20-fold in the levels of Phl p 1, Phl p 2, and Phl p 5, respectively [9]. In the case of German cockroach extracts, there was a 20-fold change in levels of Bla g 1, a massive 728-fold variation in Bla g 2, and a 12-fold difference in Bla g 5 among 12 different crude extracts [31].

The variation in the levels of significant allergens, such as Der f 1 and Der f 2, could affect the outcomes of serum and skin tests performed on dogs using different extracts, whether sourced from HDMs or other organisms. This influence of extract heterogeneity was clearly demonstrated when we tested canine sera with varying levels of IgE against Der f 1 and Der f 2 using ELISA and plates coated with the six different Der f extracts. When dogs exhibited low to moderate serum IgE levels for either of these two molecular allergens, the ELISA results for the different Der f extracts were either positive or negative. Conversely, dogs with higher sIgE levels for either of these major allergens were more likely to have positive ELISA results with all extracts. The lack of uniformity in allergen content in extracts can thus influence serological test results in dogs with allergies.

Such inconsistencies have been reported before in skin prick tests and serological testing of humans with pollen, mite, or cockroach allergies. In human allergic individuals, the likelihood of specific extracts causing either positive or negative reactions cannot be predicted, as it ultimately varies between patients. In fact, the effectiveness (i.e., potency) of an extract to detect sensitizations depends on the concentration of the various allergens present in that extract, as well as the levels of the patient’s sIgE and the allergen repertoire targeted by these sIgE.

The inconsistencies we observed in total protein content, electrophoretic migration profiles, and molecular allergen concentrations are due to the inherent difficulty in standardizing crude allergen extracts between batches and manufacturers. Indeed, the allergen composition of an extract is influenced by multiple factors, each of which is difficult to harmonize across production batches. For example, in the case of mite extracts, key sources of variability include the mite culture conditions, the type of food used to grow them, and the characteristics of the material included (for example, the proportions of different mite stages and the ratio of bodies to fecal pellets) [12]. Furthermore, variations among producers in the purification and standardization methods are additional sources of heterogeneity.

Our results argue for the need to standardize at least some characteristics of crude allergen extracts for veterinary use. As reviewed recently [12], such standardization of extracts for testing or therapeutic use (i.e., AIT) could be reached using one or more of the following approaches: the measurement of protein content, the determination of IgE reactivity and allergenic activity, the use of mass spectrometry, circular dichroism or size exclusion, the quantification of allergens by ELISA, the qualitative detection of allergens, or the determination of immunogenicity after immunization of laboratory animals [12]. Each of these methods has distinct advantages and disadvantages [12]. Unfortunately, there is no single process that can analyze all relevant attributes (physicochemical, structural, and immunological) of individual molecular components present in complex mixtures, such as crude allergen extracts [12]. As such, it is virtually impossible to standardize crude extracts, except for selected parameters (e.g., total protein content, biological activity, or concentrations of allergens X, Y, or Z).

As suggested recently [12], the standardization of allergen material for sensitization testing is likely to be best achieved with the use of purified or recombinant molecular allergens, which would also enable component-resolved diagnosis of the unique allergome for each patient. Serological (or even intracutaneous) testing with specific molecular components has the inherent advantage of being eminently reproducible. Furthermore, the use of molecular allergology allows the separation of primary from cross-reactive allergens. Finally, as recently shown, the detection of unique sensitization patterns in human patients to molecular components can help predict the likelihood of developing clinical signs of allergies or their progression [32,33,34]; currently, such studies have not been reported in animals.

The main limitation of this study lies in the absence of multiple replicates of protein and allergen concentration determination, which prevented the performance of meaningful statistical analyses. Furthermore, our results could not be compared with those of a “gold standard” extract of *Dermatophagoides farinae* containing high levels of all relevant allergens, as such a standard does not exist in human or veterinary medicine.

## 5. Conclusions

Whether we examined total protein content, protein electrophoregrams, major allergen concentrations, or the ELISA testing of sera from allergy-suspected dogs, our results confirmed the marked heterogeneity of crude HDM allergen extracts used for testing allergy-suspected pets. Such variability is likely the main cause of discrepancies seen when comparing different serological test results [35,36] or attempting to match intradermal and serological tests [37]. The use of IgE serology (and, as shown in humans, skin prick tests) with crude extracts may yield false-negative results, especially if the extract contains low levels of the allergens to which patients are hypersensitive and/or when they have low levels of IgE against these allergens. Finally, the comparison of different sensitization tests will always reveal discrepancies due to the variability in allergen profiles between the different extracts. The use of recombinant or purified allergen components and molecular-based diagnostics provides promising solutions to remedy these extract-associated inconsistencies. Further research is needed to evaluate the clinical implications of these differences between extracts, particularly their effects on intradermal and serological testing, as well as immunotherapy outcomes.

## Data Availability

All data are included in this paper.

## Supplementary Materials

The following supporting information can be downloaded at: https://www.mdpi.com/article/10.3390/vetsci12090824/s1, Table S1: PAX-derived serum levels of allergen-specific IgE against house dust mite extracts and components; Figure S1: Mass spectrometry analysis of each extract’s bands co-migrating with Der f 1 and Der f 2.

## Abbreviations

The following abbreviations are used in this manuscript:

AIT	Allergen immunotherapy
ELISA	Enzyme-linked immunosorbent assay
HDM	House dust mites
PAGE	Polyacrylamide gel electrophoresis
PAX	Pet Allergy Xplorer
SDS	Sodium dodecyl sulfate
sIgE	Specific immunoglobulin E
