# Supplementary material for: Composition Heterogeneity and Low-Molecular-Weight Allergen Content of Dermatophagoides farinae House Dust Mite Allergen Extracts Used in Veterinary Medicine
Source: Vet Sci. 2025 Aug 27;12(9):824. doi: 10.3390/vetsci12090824 (PMC12474439; doi:10.3390/vetsci12090824)
Supplement: Supplementary file 1 [file vetsci-12-00824-s001.zip › vetsci-3782451-supplementary.pdf]

Table S1: PAX-derived serum levels of allergen-specific IgE against house dust mite extracts and components

	Low levels of Der f 1-specific IgE			Medium levels of Der f 1-specific IgE			High levels of Der f 1-specific IgE			Low levels of Der f 2-specific IgE				Medium levels of Der f 2-specific IgE		High levels of Der f 2-specific IgE			No detectable Der f, Der f 1, and Der f 2 specific IgE		
Dog #	1	2	3	4	5	6	7	8	9	10	11	12	13	14	15	16	17	18	19	20	21
Der f extract	30,24	52,21	40,79	28,00	28,03	35,60	46,67	153,45	71,92	366,14	131,44	28,40	32,35	28,09	31,46	135,74	39,62	37,87	20,96	22,67	26,22
Der f 1	28,52	29,96	69,78	131,44	178,68	268,14	768,30	904,30	1293,30	21,25	24,14	23,15	21,42	22,13	21,86	22,70	22,58	22,75	20,05	22,49	22,57
Der f 2	19,17	23,39	19,89	20,85	18,12	18,52	18,44	24,45	18,09	28,28	39,56	53,42	72,24	171,25	209,57	671,12	676,19	1200,51	19,07	20,81	24,22
Der f 15	19,71	22,10	21,81	19,74	16,15	16,29	18,04	17,98	15,41	18,89	18,59	22,59	18,70	19,87	21,41	18,43	24,18	20,18	19,81	20,54	21,81
Der f 18	17,59	21,50	19,40	20,53	18,47	16,88	19,69	19,72	18,26	20,53	18,20	21,47	17,62	18,40	19,24	19,18	21,49	23,06	19,38	20,40	22,97
Der p extract	21,64	28,09	24,49	40,15	37,67	186,19	119,54	43,74	125,83	24,54	20,63	21,74	20,08	20,81	24,75	23,98	27,82	22,63	21,61	22,21	24,43
Der p 1	18,30	191,66	43,75	202,87	226,46	148,02	1158,03	838,21	1573,17	18,93	19,59	24,09	15,00	18,69	21,51	24,22	22,75	23,87	20,08	20,32	22,96
Der p 2	23,14	24,14	24,49	19,03	19,13	21,77	20,56	27,11	20,35	27,60	36,58	45,87	54,95	100,22	209,41	855,13	809,01	1198,96	19,28	21,32	22,05
Der p 5	20,90	18,05	18,70	20,74	20,21	22,11	21,01	60,89	20,74	20,34	20,97	18,61	20,70	18,91	19,46	18,91	19,31	16,74	19,18	19,05	20,66
Der p 7	23,20	23,07	22,26	23,39	22,05	23,50	22,92	20,07	20,75	22,92	21,92	22,54	21,57	20,98	21,31	21,54	23,41	23,92	21,32	19,36	22,89
Der p 10	21,96	20,21	20,05	18,79	19,98	21,92	19,47	18,63	19,54	17,63	18,18	18,34	20,02	17,98	17,55	18,94	20,16	18,47	17,29	16,60	19,28
Der p 11	20,22	21,80	20,80	19,06	18,67	30,89	20,17	34,27	20,11	21,25	18,77	21,70	18,79	19,20	21,20	19,67	20,60	21,38	19,36	20,00	20,11
Der p 20	19,42	21,98	23,06	20,08	19,29	18,67	19,59	19,92	20,65	19,51	19,88	21,43	21,23	18,02	19,64	18,76	21,03	19,16	20,18	19,31	21,96
Der p 21	23,87	24,56	23,55	21,87	20,34	272,49	710,28	417,63	21,38	20,02	21,64	23,78	22,72	100,39	23,19	22,29	58,97	22,58	19,92	21,01	24,83
Der p 23	23,60	22,94	21,37	20,54	17,98	74,59	57,45	18,13	17,96	19,23	21,44	20,40	23,01	18,91	199,95	17,44	19,02	21,27	19,06	18,17	20,93

These values represent concentrations of specific IgE in ng/mL

Note: in the PAX, Der f 15 and Der f 18 are recombinant allergens that do not express the natural glycans that cross-react with those of Toxocara canis

Colour	Meaning
(green)	low level of specific IgE (28-99,99 ng/mL) (PAX Class 1)
(yellow)	medium levels of specific IgE (100,00-399,00 ng/mL) (PAX Class 2)
(red)	high level of specific IgE (≥ 400,00 ng/mL) (PAX Class 3 and 4)

Figure S1. Mass spectrometry analysis of each extract's bands co-migrating with Der f 1 and Der f 2

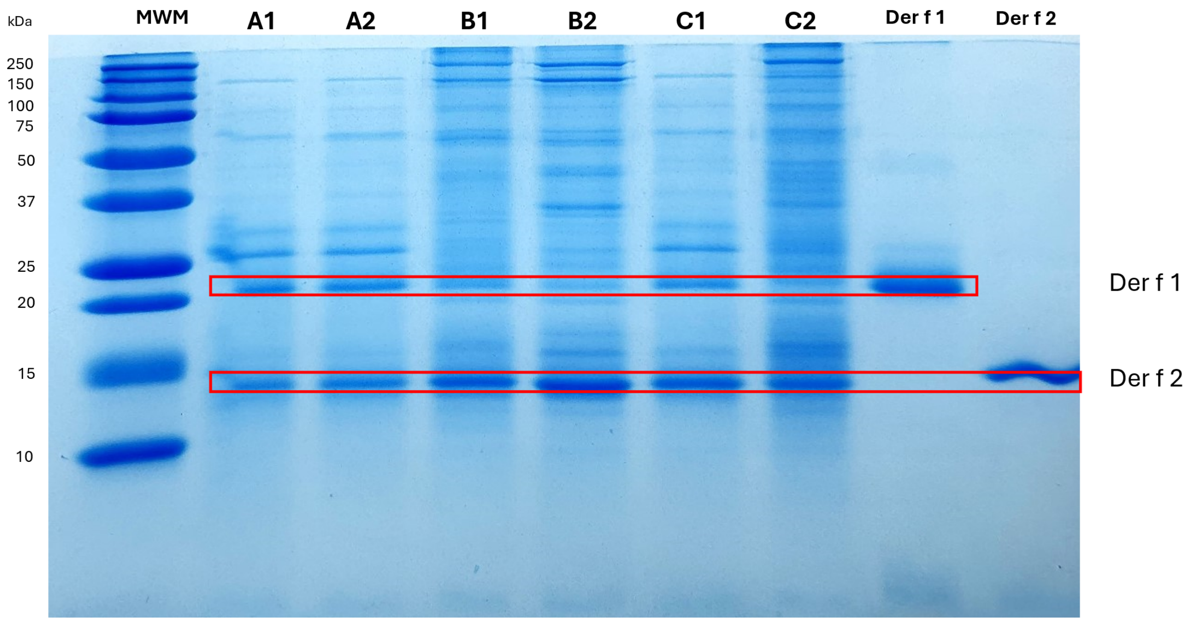

On electrophoretic gels, bands can contain multiple proteins of similar molecular weights, which might affect the assessment of the relative concentration of a protein when visually assessing bands. To address this, bands corresponding to the migration patterns of both Der f 1 and Der f 2 were cut out from each extract lane in the gel and sent for mass spectrometry analysis. The proteins in these bands underwent reduction, alkylation, and in-gel digestion with trypsin to generate peptides. The peptides were then extracted from the gel and purified using solid-phase extraction. After purification, the peptides were analysed using liquid chromatography-tandem mass spectrometry (LC-MS/MS) to ionize and fragment them and measure their mass-to-charge ratios. The resulting MS/MS spectra were searched against protein databases using MASCOT without preferred taxonomy. Finally, the results were interpreted based on MASCOT scores and expected values.

Der f 1 bands: (highest score, P value)

Extracts:

A1: **Der f 1** (410; 5e-40)

A2: **Der f 1** (195; 1.3e-18)

B1: **Der f 1** (328; 7.9e-32) **Der f 36** (90; 5.5e-8)

B2: **Der f 1** (340; 5e-33) **Der f 36** (75; 1.5e-6)

C1: **Der f 1** (410; 5e-40)

C2: **Der f 1** (131; 4e-12)

Theoretical molecular weights: Der f 1: 25 kDa; Der f 36: 23 kDa

Der f 2 bands: (highest score, P value)

Extracts:

A1: **Der f 2** (456; 1.3e-44) **Der f 34** (43; 0.0027)

A2: **Der f 2** (461; 4e-45) **Der f 34** (54; 0.00019)

B1: **Der f 2** (444; 2e-43) **Der f 34** (118; 7.9e-11)

B2: **Der f 2** (498; 7.9e-49) **Der f 34** (169; 6.3e-16)

C1: **Der f 2** (667; 1e-65) **Der f 34** (87; 9.5e-8)

C2: **Der f 2** (615; 1.6e-60) **Der f 34** (79; 6.9e-7)

Theoretical molecular weights: Der f 2: 15 kDa; Der f 34: 16 kDa
